# Influence of Temperature and Sodium Sulfate Content on the Compaction Characteristics of Cement-Stabilized Macadam Base Materials

**DOI:** 10.3390/ma13163610

**Published:** 2020-08-14

**Authors:** Ke Tang, Xue-Song Mao, Qian Wu, Jian-Xun Zhang, Wan-Jun Huang

**Affiliations:** School of Highway, Chang’an University, Middle-section of Nan’er Huan Road, Xi’an 710064, China; 2018021007@chd.edu.cn (K.T.); wuqian@chd.edu.cn (Q.W.); 2018121169@chd.edu.cn (J.-X.Z.); 2019021015@chd.edu.cn (W.-J.H.)

**Keywords:** road engineering, sulfate saline soil area, cement stabilized macadam base materials, compaction characteristics, SEM and EDS, orthogonal tests

## Abstract

This paper describes an experimental investigation into the compaction characteristics of cement-stabilized macadam base materials (CSMBM) in a saline soil area. Through the field tests, the main causes of arch expansion in an existing road were analyzed. Based on this, the compaction tests and microscopic tests were designed to analyze the impacts of temperature, sodium sulfate content and cement content on the compaction characteristics of CSMBM. Then, the orthogonal test was designed to analyze the effects of the degree of the temperature, the cement content, and the sodium sulfate content on the compaction results of the CSMBM. Feld tests results show that the temperature, sodium sulfate content and cement content may be the main causes of arch expansion. The compaction tests show that with the temperature increasing, the optimal water content (OWC) decreases, but the maximum dry density (MDD) increases; with the sodium sulfate content increasing, the OWC increases, but the MDD decreases; with the cement content increasing, both MDD and OWC increase. The microscopic tests show that the increase of temperature and cement content is beneficial to the compactness between cementitious materials and aggregates, while the increase of sodium sulfate content makes the whole structure of cementitious materials and aggregates increasingly rough. The orthogonal test shows that the temperature has the greatest influence on the MMD, and the sodium sulfate content has the greatest influence on the OWC. Thus, in a sulfate saline soil area, the construction temperature, the sodium sulfate content and the cement content should be controlled to ensure the compaction quality of CSMBM.

## 1. Introduction

In the area of Ejina, Inner Mongolia, China, there is a special form of pavement disease on many roads, named the arch expansion of the pavement [1,2]. Most previous study showed that the arch expansion mainly occurs in the subgrade structure layer due to the existence of sodium sulfate and variation temperature [3,4,5]. The sodium sulfate can exist in three forms: sodium sulfate solution, thenardite, mirabilite (Na_2_SO_4_·10H_2_O), which was caused by the content of sodium sulfate and environment temperature. When it was at temperature below 32.4 °C, the Na_2_SO_4_·10H_2_O is the only stable phase; when it was at temperature above 32.4 °C, the thenardite is the only stable phase [6,7,8,9]. Lai et al. [5] and Bing et al. [10] and Zhang et al. [11] thought that a ratio of molar volume change for Na_2_SO_4_·10H_2_O particle is 3.14 times and the transformation between thenardite and Na_2_SO_4_·10H_2_O is dependent on temperature. Therefore, the environmental temperature during construction may have a great impact on the quality of subgrade construction in sulfate saline soil area, especially the compaction quality.

A series of laboratory tests were carried out by Kampala et al. [12], to examine compaction characteristics and strength development of reconstituted sulfate-bearing soil-cement. Wen et al. [13] found that the maximum dry density (MDD) and optimal water content (OWC) are affected by the three states of sodium sulfate (sodium sulfate solution, thenardite, and Na_2_SO_4_·10H_2_O) and the relative content among them, and they consider the influence of false drying phenomenon on OWC. Wen et al. [14] also found that the compaction characteristics and microstructure characteristics of sulfate saline soil are closely related to the state of sodium sulfate in the soil. As for the cement-stabilized macadam base materials (CSMBM) in a sulfate saline soil area, its compaction quality is also determined by the OWC and MDD, which were influenced by the cement, sodium sulfate and environment temperature. Chhorn et al. [15] mentioned that dry density was used to characterise the compactibility of roller compacted concrete, which is affected easily by various factors such as aggregates gradation and water content. Odumade et al. [16] through tests found that the MDD increases and OWC decreases as the cement content increases. Han et al. [17] used the isothermal calorimeter to test the cement hydration rate, and found that the cement hydration rate is obviously fast at high temperature. Wang et al. [18] found that under the effect of high temperatures, the hydration rate of the cement increases, resulting in more C–S–H and C–A–H gels, which bridge the aggregate tightly together, thus increasing the compactness of the CSMBM.

There are many researches on the influence of sodium sulfate on cement-based materials, which mainly focused on cement concrete. Sodium sulfate reduced the properties of concrete by reacting with cement hydration products to form ettringite and gypsum, which can be explained by the Equations (1) and (2) [19,20,21].

(1) $$\begin{array}{l} {3(\mathrm{CaSO}}_{4}\cdot \left(H_{2}O\right)+4\mathrm{CaO}\cdot {\mathrm{Al}}_{2}O_{3}\cdot {12H}_{2}{O+14H}_{2}O\to \\ 3\mathrm{CaO}\cdot {\mathrm{Al}}_{2}O_{3}\cdot {3\mathrm{CaSO}}_{4}\cdot {31H}_{2}O+\mathrm{Ca}{\left(\mathrm{OH}\right)}_{2} \end{array}$$

(2) $$\mathrm{Ca}{\left(\mathrm{OH}\right)}_{2}{+\mathrm{SO}}_{4}^{2-}{+2H}_{2}O\to {\mathrm{CaSO}}_{4}\cdot {32H}_{2}{O+2\mathrm{OH}}^{-}$$

However, few studies took sodium sulfate, cement and temperature into account to study the compaction characteristics of cement-stabilized macadam base material in a sulfate saline soil area.

This paper is divided into three parts. Through the observation and the chemical titration test of the cement-stabilized macadam material in the arch expansion road section, the ingredient and content of the soluble salt is determined. Through the test of compaction, scanning electronic microscopy (SEM) and energy-dispersive spectroscopy (EDS), the influences of the cement content, sodium sulfate content and temperature variation on the compaction characteristics of cement stabilized macadam in sulfate soil area were analyzed. Through the orthogonal tests scheme with three factors and three levels, the effects degree of the cement content, sodium sulfate content, temperature on the OWC and MDD of the cement-stabilized macadam base material in sulfate soil area were analyzed. The obtained results are helpful to ensure the compaction quality of CSMBM in sulfate saline soil area.

## 2. The Causes of the Arch Expansion Section

### 2.1. Excavation of the Arch Expansion Pavement

**Figure 1:**
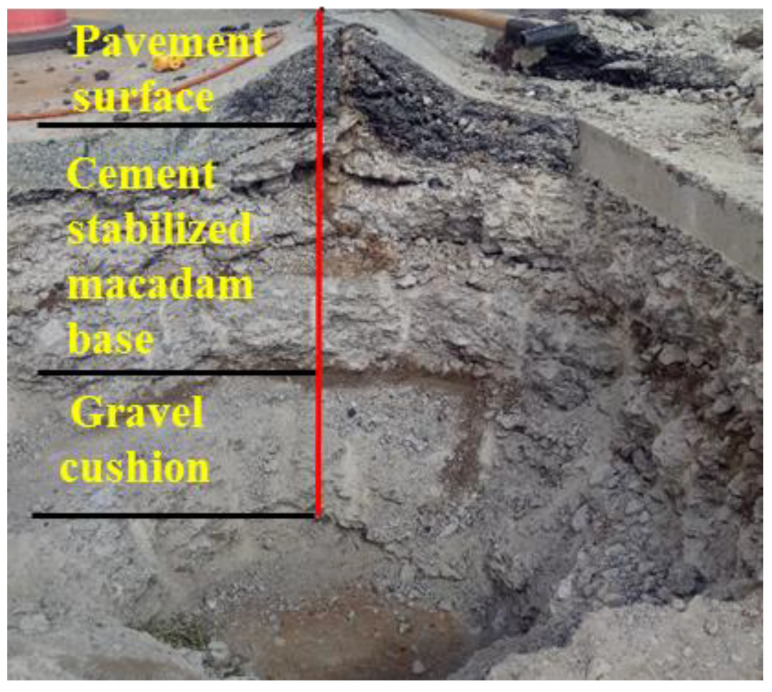
Pavement structure at the arch expansion section.

In order to know the causes of the arch expansion of the pavement in Ejina, Inner Mongolia, China, the writer and worker excavated the most seriously arch expansion of the road shown in Figure 1.

As shown in Figure 1, the arch expansion mainly occurs in the pavement surface and cement-stabilized macadam base. It is worth noting that there is no arch expansion phenomenon in the subgrade. The arch expansion of asphalt pavement is easily affected by that of base. This is similar to the study by Titus-Glover et al. [22] and Gao et al. [23], who have mentioned that the cement-stabilized macadam base can develop fatigue and transverse shrinkage cracks, which ultimately propagate through the asphalt pavement surface.

### 2.2. The Salt Content in the Arch Expansion Section

**Figure 2:**
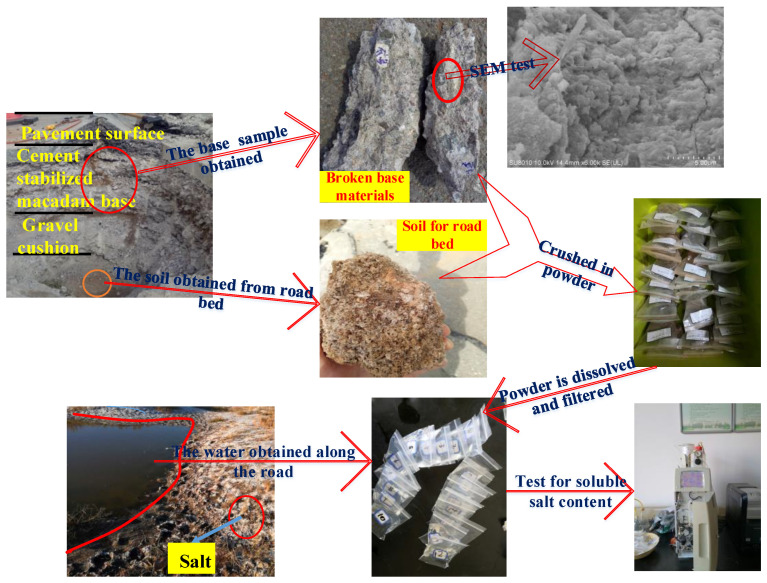
The details of the salt content of the test process.

In order to clarify the inner mechanic of arch expansion of cement stabilized macadam base, the content of soluble salt, SO_4_^2−^ ion, and Cl^−^ ion in five kinds of subgrade filler, four kinds of CSMBM and three kinds water were tested by the methods of chemical titration and oven drying [20]. The details of the test process are shown in Figure 2, and the test results are shown in Table 1, Table 2 and Table 3.

As shown in Table 1, the soluble salt content of all subgrade filler samples is more than 0.3%, and the ratios of $C\left({\mathrm{Cl}}^{-}\right)/2C\left({\mathrm{SO}}_{4}^{2-}\right)$ is less than 0.3. Therefore, the subgrade filler is sulfate saline soil. Table 2 shows that the soluble salt content of all samples are less than 0.3%, but the ratios of $C\left({\mathrm{Cl}}^{-}\right)/2C\left({\mathrm{SO}}_{4}^{2-}\right)$ are still less than 0.3. This indicates that Cl^−^ ion cannot inhibit the reaction of SO_4_^2^^−^ ion with hydration products [24]. From the Table 3, there are three kinds of water: Fresh water, brackish water, salt water. Besides, the content of SO_4_^2^^−^ ion is much higher than that of chloride ion. Therefore, it is very important to control the water quality when mixing and curing CSMBM.

### 2.3. The Local Environment Temperature

**Figure 3:**
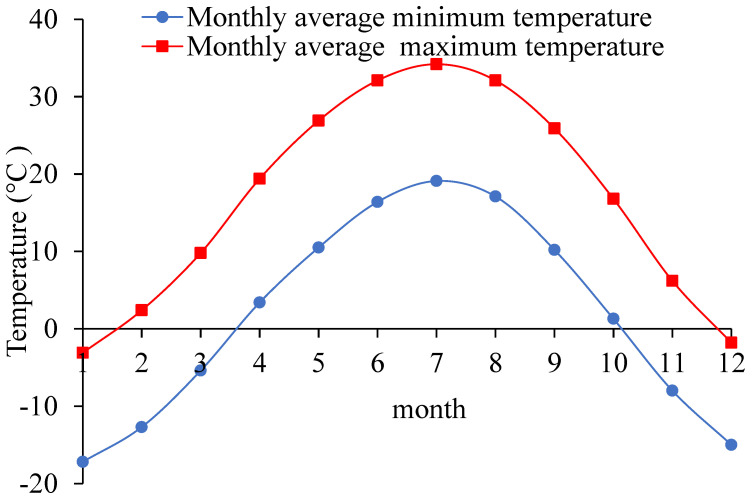
The maximum temperature and the minimum temperature each month.

Through consulting meteorological data, the monthly average maximum temperature and average minimum temperature in Ejina, Inner Mongolia, China in recent ten years were statistically sorted out as shown in Figure 3.

Figure 3 shows that this area has a typical climate. There is a large difference (about 20–25 °C) between monthly average maximum temperature and the monthly average minimum temperature each month in the last 10 years. The form of the sodium sulfate and the hydration reaction rate of the cement are affected by the temperature easily [8,9,17].

Through the analysis above, before the CSMBM was constructed in sulfate saline soil area, it is necessary to discuss the influence of temperature, cement, sodium sulfate content on the compaction quality of CSMBM.

## 3. Test Materials and Method

### 3.1. Test Materials

**Figure 4:**
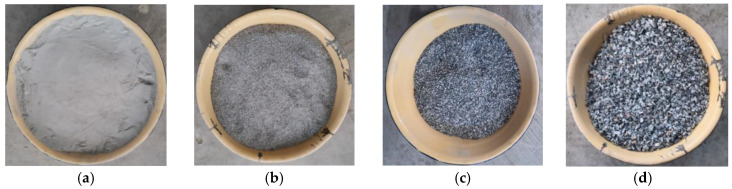

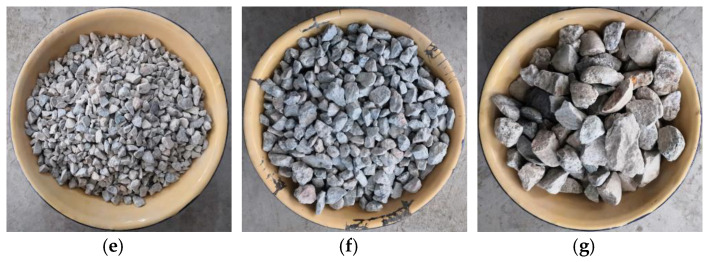
The aggregates screen size: (**a**) 0~0.075 mm, (**b**) 0.075~0.6 mm, (**c**) 0.6~2.36 mm, (**d**) 2.36~4.75 mm, (**e**) 4.75~9.5 mm, (**f**) 9.5~19 mm, (**g**) 19~31.5 mm.

1. The cement used in the experimental is 42.5R ordinary Portland cement.
2. In this paper, the aggregates are based on the mix ratio of G7 road. The aggregates used was in line with the “Road Pavement Construction Technical Specifications (JTJ034-2000)” [25] requirements. The aggregates screen size is shown in Figure 4. The aggregate percent passing is shown in Table 4.
3. Tap water was used for mixing water, and the tap water’s indexes were tested according to “JGJ 63-2006 Stander for water in concrete” [26]. The results are shown in Table 5.
4. Through the observation and the chemical titration test, there is a certain amount of SO_4_^2−^ in CSMBM. So, the sodium sulfate was used as the salt for the tests.

### 3.2. Tests Design

#### 3.2.1. Test Scheme Design

In order to study the compaction characteristics of the CSMBM in sulfate saline soil area, the compaction test, the SEM tests and the EDS tests were carried out by controlling variable method. The SEM tests were carried out using a Hitachi S-8100 scanning electron microscope (Tokyo, Japan). The EDS tests were carried out using an Oxford Electronic Differential System (Oxford, United Kingdom). The specific schemes of these tests are shown in Table 6. It is noted that the amount of sodium sulfate, cement, and water is based on the percentage of aggregates according to the literature [27]. According to Figure 3, in the area of Ejina, Inner Mongolia, China, the monthly average maximum temperature at the beginning and the end of the year in the last 10 years was lower than 0 °C, which is not conducive to the construction of subgrade and pavement. Therefore, the construction of subgrade and pavement in this area mainly starts in March and ends in November. During this time, the temperature is about 10–35 °C.

#### 3.2.2. Compaction Tests

According to the “Road Pavement Construction Technical Specifications (JTJ034-2000)” [25], wet mix method was used to prepare the CSMBM in sulfate saline soil area [14]. The details of wet mix are as follows. Firstly, according to Table 6, the sodium sulfate was dissolved in water, at the same time, the glass rod was used to stir the sodium sulfate to dissolve fully. Secondly, the sodium sulfate solution and aggregates were thoroughly stirred in a plastic container. Finally, it was placed for no more than 2 h to avoid the CSMBM caking caused by sodium sulfate. In order to reduce the influence of time on compaction test results, the mixing time is the same when cement was added. After 20 min, the heavy compaction tests were carried out.

A standard heavy compaction test method was adopted [16,27]. The methodology involved using a mould with 152 mm diameter and 120 mm height, which has a volume of 2177 cm^3^, a hammer weighing 4.5 kg with 50 mm diameter. Firstly, the mass of empty mould was measured to be M_0_, which is accurate to 0.001 kg. The cement-stabilized macadam material prepared was placed in three layers of about equal thickness and each layer is subjected to 98 blows from the hammer by falling freely through a distance of 450 mm in the mould. The mass of compacted sample with the mould was measured to be M_1_, which is accurate to 0.001 kg, and part of it was taken from the center to oven dry at 110 °C for 10 h for the purpose of determining the water content. The dry density can be calculated by Equations (3) and (4)

(3) $$\rho =\left(M_{1}-M_{0}\right)/V$$

where: ρ is the wet density of the cement stabilized macadam base material (g/cm^3^); V is the volume of the mould (cm^3^).

(4) $$\rho_{d}=\rho /\left(1+0.01w\right)$$

where: $\rho_{d}$ is the dry density (g/cm^3^); w is water content.

The dry density is regarded as the ordinate and the water content as the abscissa. The curve is fitted by the quadratic curve method. The peak point of the curve is the MMD and the OWC.

#### 3.2.3. Microstructure Tests

After the compaction tests, 9 groups of OWC and MDD will be obtained. The compaction tests were carried out again according to the value of OWC and MDD. Then the compacted samples cut into specimens of 5 mm × 5 mm × 5 mm by cutting machine were placed in the high- and low-temperature test chamber for SEM tests, where the temperature was set at 10 °C, 25 °C and 35 °C respectively. It is note that the SEM are mainly to observe the morphology of cementitious materials. Before tests, a layer of gold was plated onto the specimen surface to prevent charge accumulation on the surface. Due to the change of sodium sulfate content, the EDS was used to test the change in mineral phases.

### 3.3. Orthogonal Test Design

Orthogonal test can reduce the number of experiments greatly. In this test, the orthogonal tests with three factors and three levels [28] was used to carry out range analysis, so as to analyze the effects degree of the cement content, sodium sulfate content, temperature on the OWC and MDD of the CSMBM in sulfate saline soil area. The levels of each factor are shown in Table 7 The mass of each material is shown in Table 8.

Range analysis: the theories of range analysis are as in Equations (5) and (6),

(5) $$L_{ij}=\frac{\sum_{m=1}^{n}k_{ij,m}}{n}$$

where $L_{ij}$ is the average value of the test result of factor i at the level *j*. $k_{ij,m}$ is the mth n result of factor *i* at the level *j*. *n* is the number of calculation results of factor *i* at the level *j*.

(6) $$R_{i}=\max\;\left\{L_{ij}\right\}-\min\left\{L_{ij}\right\}$$

Where $R_{i}$ is the difference between the maximum and minimum $L_{iJ}$ at each factor.

## 4. Test Results and Discussion

### 4.1. Influence of Temperature on the Form of Sodium Sulfate

**Figure 5:**
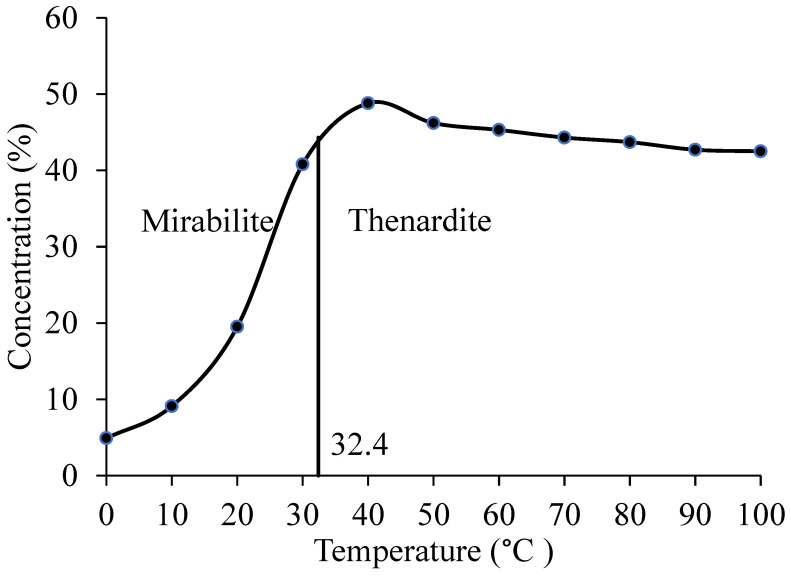
Solubility of thenardite and mirabilite versus temperature (The data digitized from Steiger and Asmussen, 2008) [29].

The solubility of sodium sulfate in water will be changed due to environmental temperature variation shown in Figure 5.

**Figure 6:**
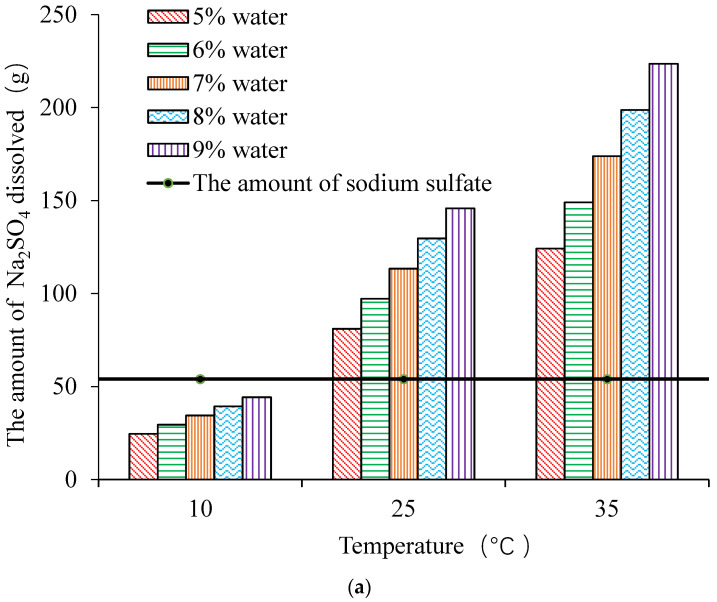

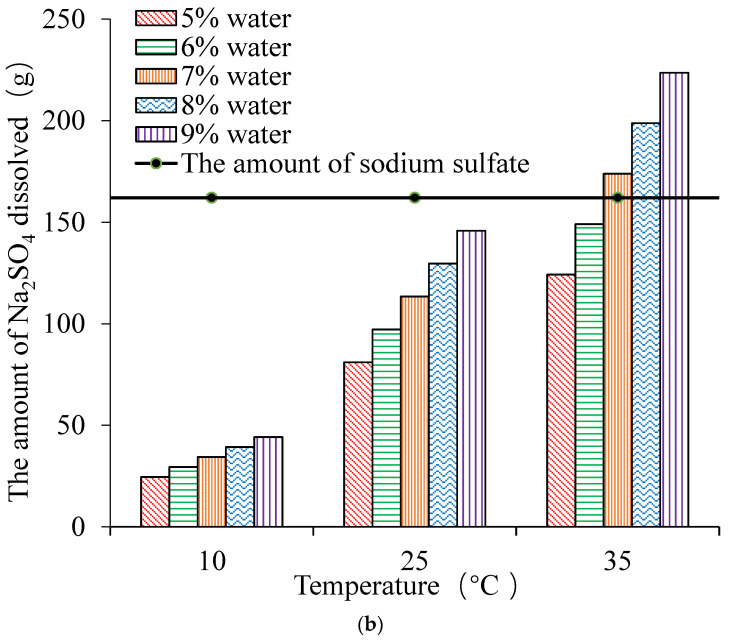
Effect of temperature on the amount of sodium sulfate dissolved in water. (**a**) 1% sodium sulfate. (**b**) 3% sodium sulfate.

Figure 5, shows that when it is 32.4 °C, the undissolved sodium sulfate exists in the form of thenardite and mirabilite, when it is above 32.4 °C; the thenardite is the only stable phase, and the solubility of thenardite increases with the decrease of temperature when it was below 32.4 °C; the mirabilite is the only stable phase, and the solubility of mirabilite increases with the increase of temperature. When the content of sodium sulfate is 0%, 1% and 3%, and the temperature is 10 °C, 25 °C and 35 °C, the dissolution of sodium sulfate is shown in Figure 6 [30].

As shown in Figure 6, when the water content and the sodium sulfate content are constant, and the temperature increases from 10 °C to 35 °C, the amount of sodium sulfate dissolved increases by 350%. This is consistent with the solubility of sodium sulfate changes with temperature in Figure 5. It can also be seen from Figure 6a when it is 10 °C, the sodium sulfate can not be dissolved completely. So there are both sodium sulfate solution and Na_2_SO_4_·10H_2_O. However, when it is 25 °C, 35 °C, there is only sodium sulfate solution. At the same time, it can be found from Figure 6b when the content of sodium sulfate is 3% that there are both sodium sulfate solution and Na_2_SO_4_·10H_2_O at 10 °C and 25 °C, while there are both sodium sulfate solution and thenardite at 35 °C. This is mainly due to that when it is lower than 32.4 °C, the sodium sulfate undissolved is in the form of Na_2_SO_4_·10H_2_O, and when it is more than 32.4 °C, the sodium sulfate undissolved is in the form of thenardite [7,31].

### 4.2. Influence of Temperature on the Compaction Characteristics

#### 4.2.1. The Compaction Results

**Figure 7:**
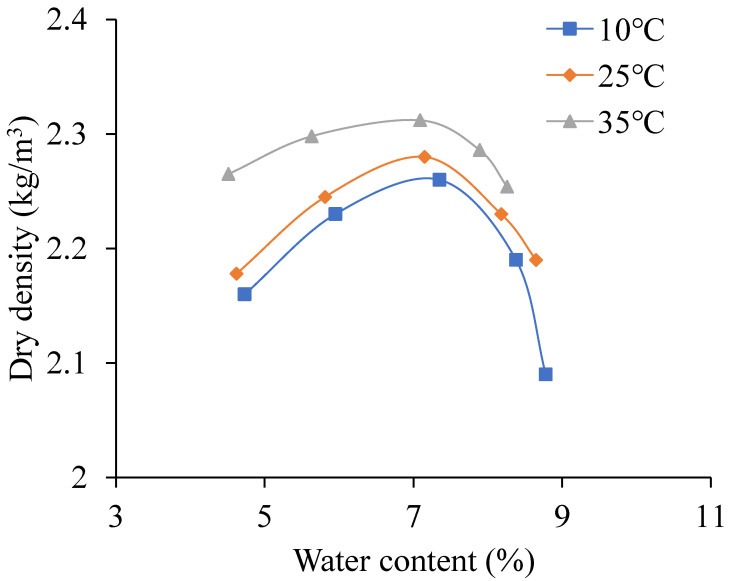
The relationship curves between water content and dry density.

**Figure 8:**
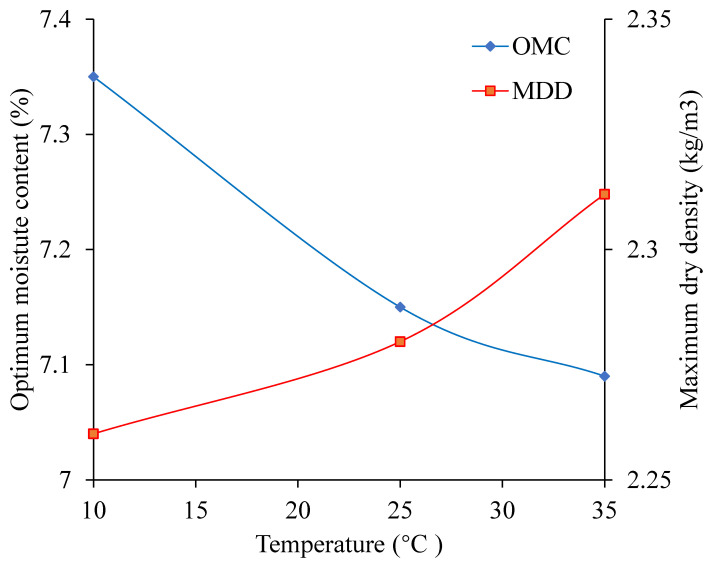
The influence of temperature on the compaction results.

When the cement content is 3%, the sodium sulfate is 3%, and the temperature changes from 10 °C to 35 °C the relationship curves between water content and dry density is shown in Figure 7. The OWC and MDD change with temperature as shown in Figure 8.

As shown in Figure 7, with the water content increasing, the dry density increases first and then decreases. It also can be seen that the higher the temperature is, the bigger the dry density.

As shown in Figure 8, with the temperature increasing, the OWC decreases, and the MDD increases. When the temperature increases from 10 °C to 35 °C, the MDD increases by 0.052 kg/m^3^ and the OWC decreases by 0.26%. This is mainly because the increasing temperature leads to the content of sodium sulfate solution increasing, which reduces the friction between the aggregates, makes the aggregates cemented tightly [32,33,34]. In this condition, the CSMBM can be compacted at low water content. This indicates that when the water content is certain, with the temperature increasing, the content of sodium sulfate solution is increasing, and the CSMBM can be compacted at low water content. So with the temperature increasing, the OWC decreases and the MDD increases.

It can also be seen from Figure 8 that when the temperature increases from 10 °C to 25 °C, the MDD increases by 0.8% and the OWC decreases by 0.2%, and when the temperature increases from 20 °C to 35 °C, the MDD increases by 1.4% and the OWC decreases by 0.06%. This is mainly because when it is lower than 32.4 °C, sodium sulfate is in the form of sodium sulfate solution and Na_2_SO_4_·10H_2_O, and when it is more than 32.4 °C, sodium sulfate is in the form of sodium sulfate solution and thenardite. Compared with Na_2_SO_4_·10H_2_O, thenardite is less than 10 molecules of water, which indicates that 10 molecules of water are removed after the Na_2_SO_4_·10H_2_O is dried at oven. So, according to the Equations (3) and (4), when the temperature increases from 10 °C to 25 °C, the amount of MDD increasing is less, and the amount of the OWC increasing is much. By contrast, when the temperature increases from 20 °C to 35 °C, the amount of MDD increasing is much, and the amount of the OWC increasing is less. This finding is similar to the research by Wen et al. [13,14].

Therefore, the construction temperature will affect the solubility of sodium sulfate to change the range of the OWC and the MDD, and ultimately affects the compaction quality of CSMBM. Given this, it is necessary to choose a reasonable construction temperature.

#### 4.2.2. Microscopic Tests Results

**Figure 9:**
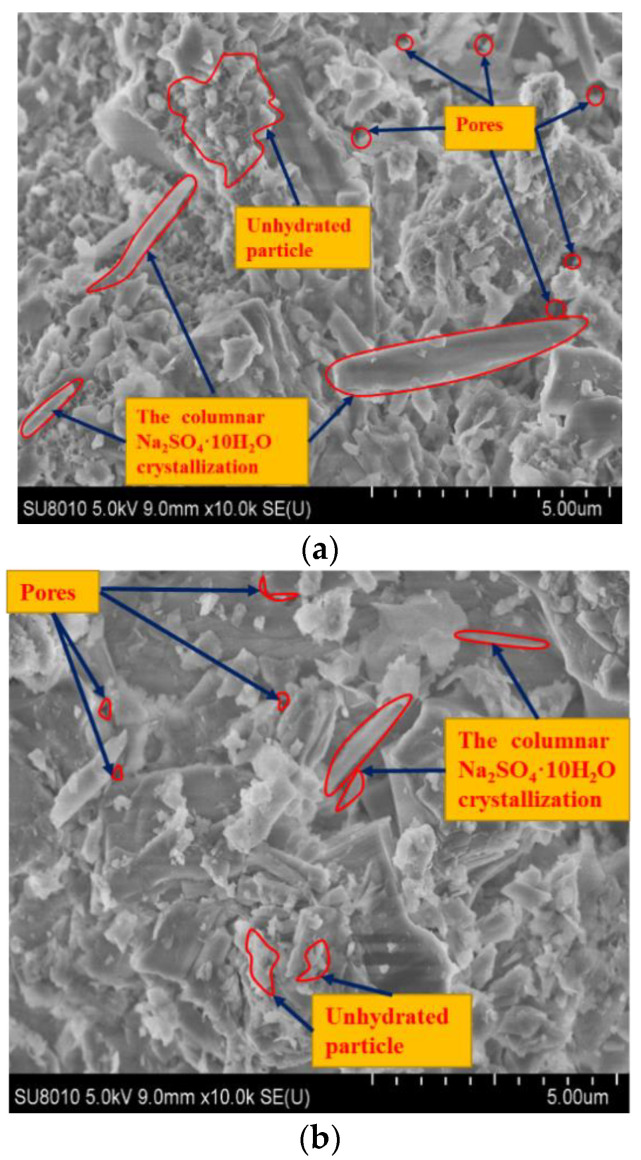

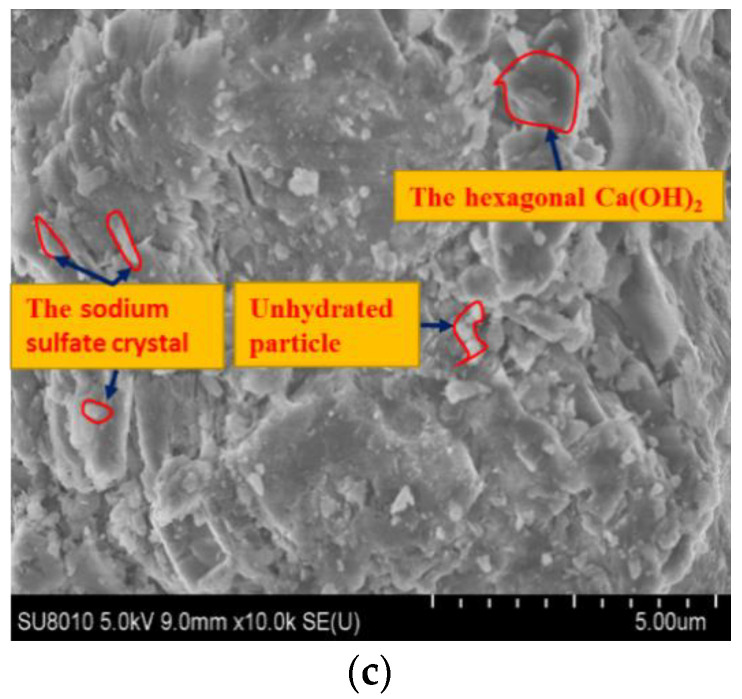
Scanning electron microscopy (SEM) image of specimens with different temperature. (**a**) Cement: 3%, sodium sulfate: 3%, temperature: 10 °C. (**b**) Cement: 3%, sodium sulfate: 3%, temperature: 20 °C. (**c**) Cement: 3%, sodium sulfate: 3%, temperature: 35 °C.

The results of SEM test are shown in Figure 9.

As shown in Figure 9, with the temperature increasing, the compactness of the structure composed of aggregates and cementitious material is gradually improving. When it is 10 °C, there is a lot of columnar Na_2_SO_4_·10H_2_O crystallization and some unhydrated particles in specimens, and the cementitious material has no obvious layer structure but has the biggest pore content, shown in Figure 9a. When it is at 20 °C, there is some columnar Na_2_SO_4_·10H_2_O crystallization and a little unhydrated particle in specimens, and the cementitious material is mainly in the form of incomplete layer with poor order and some pores, as shown in Figure 9b. When it is at 35 °C, there is some Ca (OH)_2_ and sodium sulfate crystal in the specimens and the cementitious material is mainly in the form of thin layer with smooth surface, good order and less pore content, shown in Figure 9c. 

There are two reasons for this phenomenon. Firstly, when it is at 35 °C (>32.4 °C), both the solubility of the sodium sulfate and the sodium sulfate solution content are the highest, which has a lubrication effect on the compaction test and helps the structure of the CSMBM to be more dense. Secondly, when the temperatures are at 25 °C, 10 °C (<32.4 °C), both the solubility of the sodium sulfate and the sodium sulfate solution content decrease, but the Na_2_SO_4_·10H_2_O content increases [1]. The columnar Na_2_SO_4_·10H_2_O crystallization arranged irregularly in the aggregates and cementitious material leads to this being difficult to compact.

### 4.3. Influence of Sodium Sulfate Content on the Compaction Characteristics

#### 4.3.1. The Unreal Dry Phenomenon

**Figure 10:**
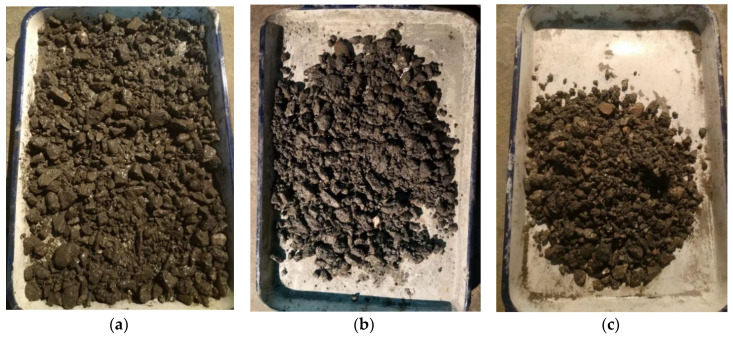
The unreal dry phenomenon in mixing the cement stabilized macadam materials. (**a**) 0% sodium sulfate. (**b**) 1% sodium sulfate. (**c**) 3% sodium sulfate.

When the cement content is 3%, the temperature is 10 °C, and the sodium sulfate content changes from 0 to 3%, the wetness degree and cohesion of the CSMBM changes, as shown in Figure 10.

As shown in Figure 10, with the sodium sulfate content increasing, the wetness degree and cohesion of the material decrease. This is mainly due to some water absorbed by sodium sulfate to form Na_2_SO_4_·10H_2_O crystallization, so that the amount of bound water is improved [8,35]. By contrast, when there is no sodium sulfate in the CSMBM, the wetness degree and cohesion of the material are the highest. This is mainly due to that water only reacting with cement to form cementitious material, which makes the CSMBM present good wetness shown in Figure 10a.

#### 4.3.2. The Compaction Results

**Figure 11:**
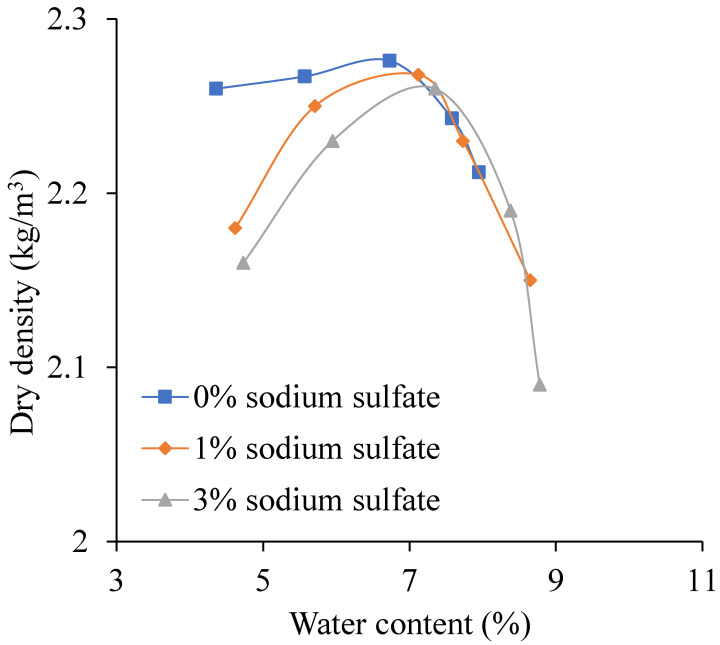
The relationship curves between water content and dry density.

**Figure 12:**
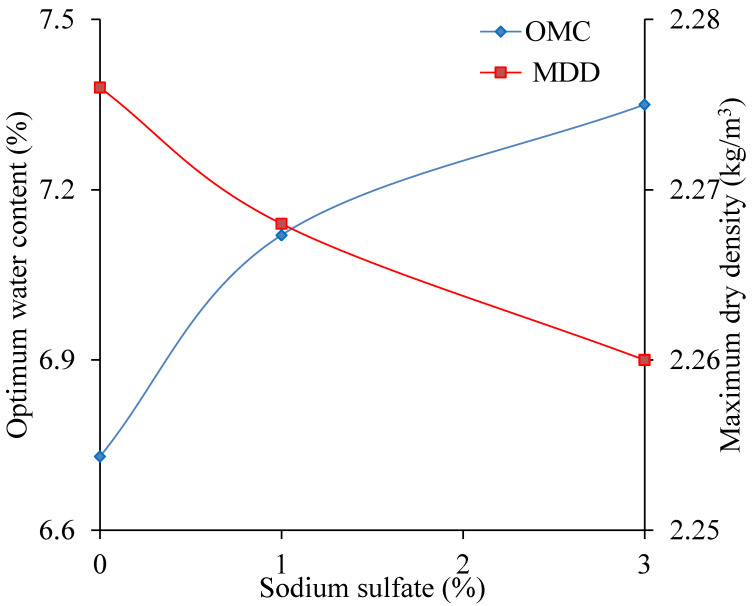
The influence of the sodium sulfate content on the compaction results.

When the sodium sulfate content changes 0% to 3%, the relationship curves between water content and dry density is shown in Figure 11. The OWC and MDD change with sodium sulfate content shown in Figure 12.

As shown in Figure 11, with the water content increasing, the dry density increases first and then decreases. It also can be seen that, the higher the sodium sulfate content, the bigger the water content.

As shown in Figure 12, with the sodium sulfate content increasing, the OWC increases, and the MDD decreases. When the sodium sulfate content increases from 0% to 3%, the MDD decreases by 0.016 kg/m^3^ and the OWC increased by 0.62%. This is mainly because when it is 10 °C (<32.4 °C), the solubility of the sodium sulfate is invariable, but the content of Na_2_SO_4_·10H_2_O is improved, which makes the OWC increase, and the MDD decrease. This phenomenon is surprising, because both in observation and compaction, the water content of cement stabilized macadam materials containing 3% sodium sulfate was obviously smaller than that of containing 0 or 1% sodium sulfate. This phenomenon has been found in the research conducted by Wen et al. [13]. They regarded it as an unreal dry phenomenon.

#### 4.3.3. Microscopic Tests Results

**Figure 13:**
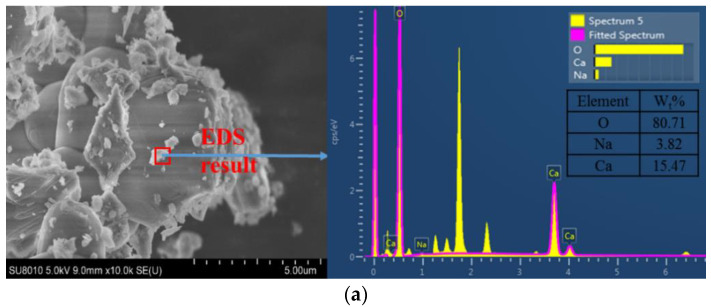

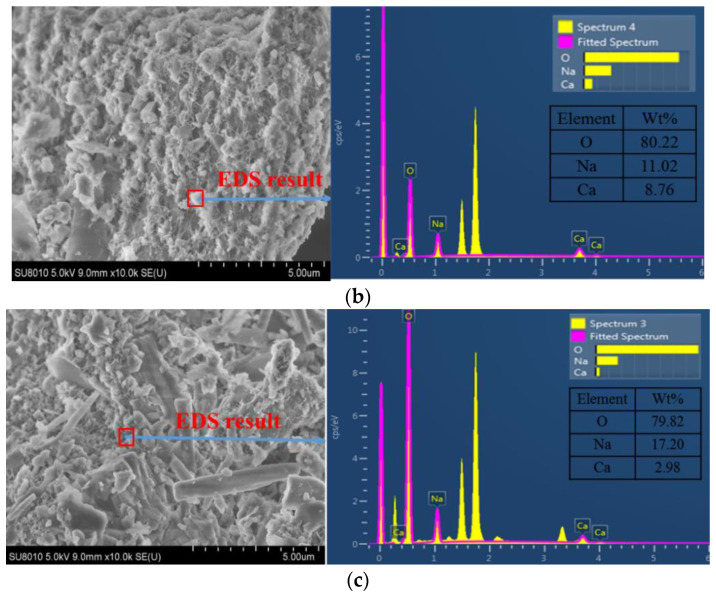
SEM images and corresponding energy-dispersive spectroscopy (EDS) results of specimens with different Sodium sulfate content. (**a**) Cement: 3%, sodium sulfate: 0%, temperature: 10 °C. (**b**) Cement: 3%, sodium sulfate: 1%, temperature: 10 °C. (**c**) Cement: 3%, sodium sulfate: 3%, temperature: 10 °C.

The results of SEM and EDS tests are shown in Figure 13.

As shown in Figure 13, by analysis of SEM images, with the sodium sulfate content increasing, the morphology and structure of cementitious materials are rougher and rougher. By analysis of the EDS results, the content of O element is stable and the content of Na and Ca element are increasing and decreasing, respectively.

As shown in Figure 13a, when the sodium sulfate content is 0%, the cementitious material is mainly in the form of a layer with dense structure and less pore content, and the content of Na and Ca are 3.82%, 15.47%, respectively. This is mainly due to the content of Ca being much higher than that of Na in hydration products and without the sodium sulfate, the hydration can be carried normally, which is beneficial to keep the structure dense [36]. As shown in Figure 13b, when the sodium sulfate content is 1%, there is a little columnar Na_2_SO_4_·10H_2_O crystallization in specimens, which makes the layer structure of cementitious material incomplete. In addition, the content of Na and Ca are 7.02%, 12.76%. This is mainly because a certain amount of sodium sulfate not only increases the content of the Na element, but also promotes the hydration reaction to form Ca [21,37]. As shown in Figure 13c, when the sodium sulfate content is 3%, the incomplete layer structure is inserted by a lot of columnar Na_2_SO_4_·10H_2_O crystallization in specimens, which wraps the cement and inhibits the hydration. So, the content of Na and Ca are 17.20%, 2.98%.

### 4.4. Influence of Cement Content on the Compaction Characteristics

#### 4.4.1. The Compaction Results

**Figure 14:**
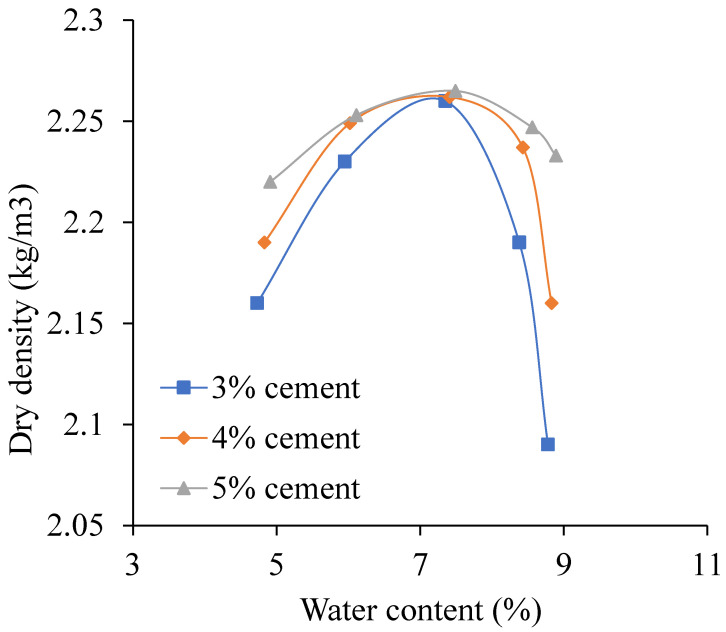
The relationship curves between water content and dry density.

**Figure 15:**
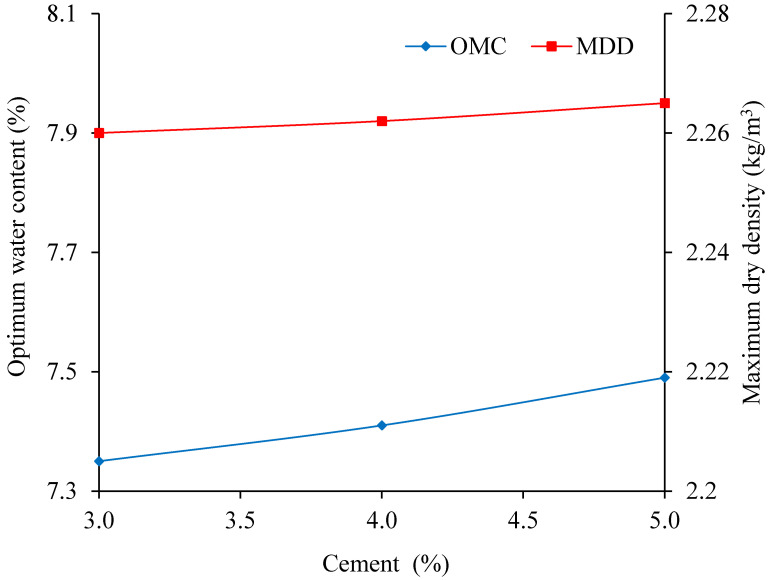
The influence of the cement content on the compaction results.

When the temperature is 10 °C, the sodium sulfate is 3%, and the cement content changes from 3% to 5%, the relationship curves between water content and dry density is shown in Figure 14. The OWC and MDD change with temperature as shown in Figure 15.

As shown in Figure 14, with the water content increasing, the dry density increases first and then decreases. It also can be seen that, the higher the cement content, the bigger the dry density.

As shown in Figure 15, with the cement increasing, the MDD and the OWC increase by 0.005 kg/m^3^ and 0.14%. There are two reasons to explain this phenomenon. Firstly, the cement content increasing accelerates the formation of hydration reaction products, which fills the pores of the aggregates. Secondly, the hydration reaction products react with the dissolved sodium sulfate to form ettringite, and gypsum, which can also fill the pores [21,37]. So, with the cement increasing, the MDD and the OWC increase.

#### 4.4.2. Microscopic Tests Results

**Figure 16:**
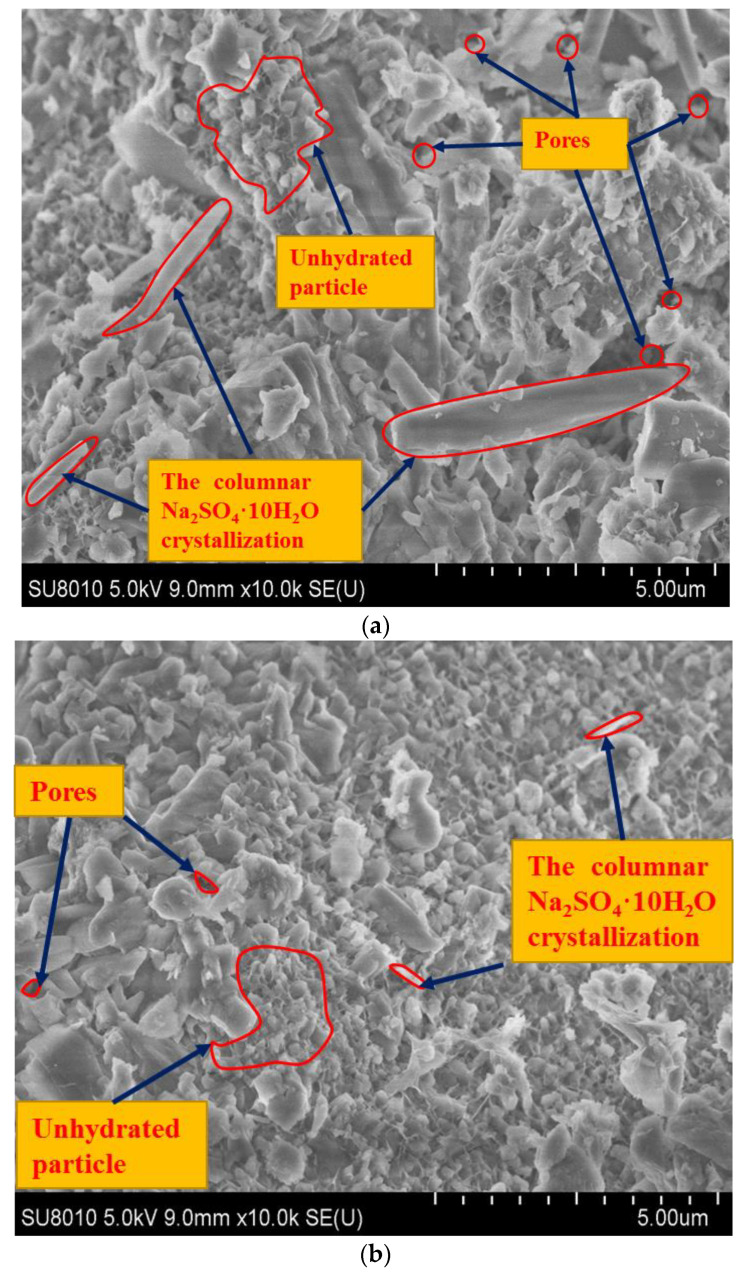

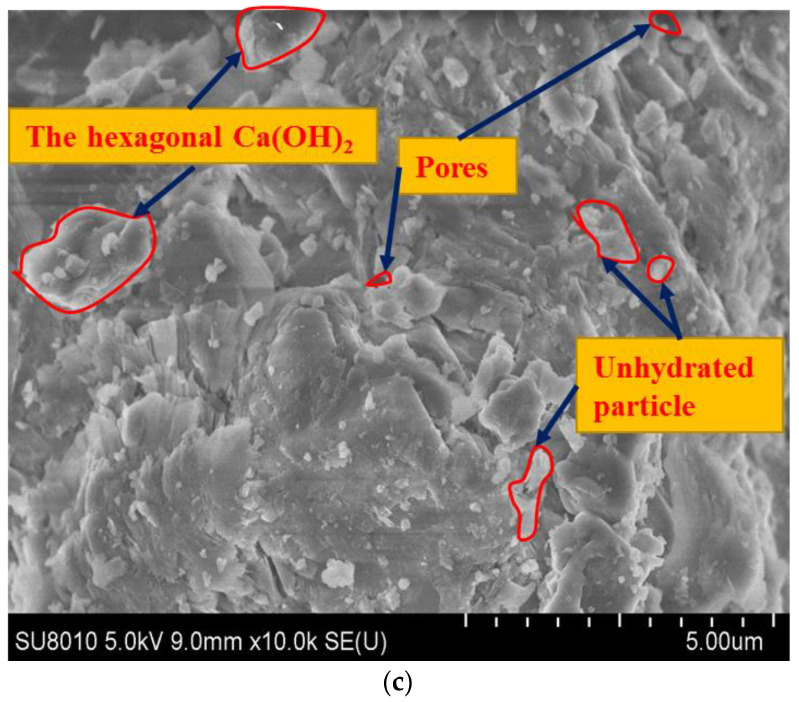
SEM images of specimens with different cement content. (**a**) Cement: 3%, sodium sulfate: 3%, temperature: 10 °C. (**b**) Cement: 4%, sodium sulfate: 3%, temperature: 10 °C. (**c**) Cement: 5%, sodium sulfate: 3%, temperature: 10 °C.

The results of the SEM tests are shown in Figure 16.

As shown in Figure 16, with the cement content increasing, the number of columnar Na_2_SO_4_·10H_2_O crystallizations and pores in the specimens decreases, and the compactness of the structure is improving gradually. On the one hand, when the cement is low (3%), there is little hydration reaction product provided to react with the SO_4_^2−^, and the sodium sulfate left is in the form of Na_2_SO_4_·10H_2_O. The columnar Na_2_SO_4_·10H_2_O crystallization arranged irregularly in the aggregates and cementitious material leads to the bad compactness shown in Figure 16a. On the other hand, when the cement is high (5%), there are some unhydrated particles, and a lot of hydration reaction product has improved to react with the SO_4_^2−^, and so there is a little sodium sulfate left to form Na_2_SO_4_·10H_2_O, and the compactness is the best, as shown in Figure 16c. 

### 4.5. Orthogonal Test Results

The OWC and MDD were obtained from the orthogonal test. The compaction test results are presented in Table 9. The range analysis results are shown in Table 10.

As shown in Table 10, the following can be obtained: (1) for the MDD of the cement-stabilized macadam materials, the order of the influence factors is as follows: A (temperature) = 0.058 > B (sodium sulfate) = 0.017 > C (cement) = 0.005; (2) for the OWC of cement-stabilized macadam materials, the order of influence factors is as follows: B = 0.30 > C = 0.26 > A = 0.10.

**Figure 17:**
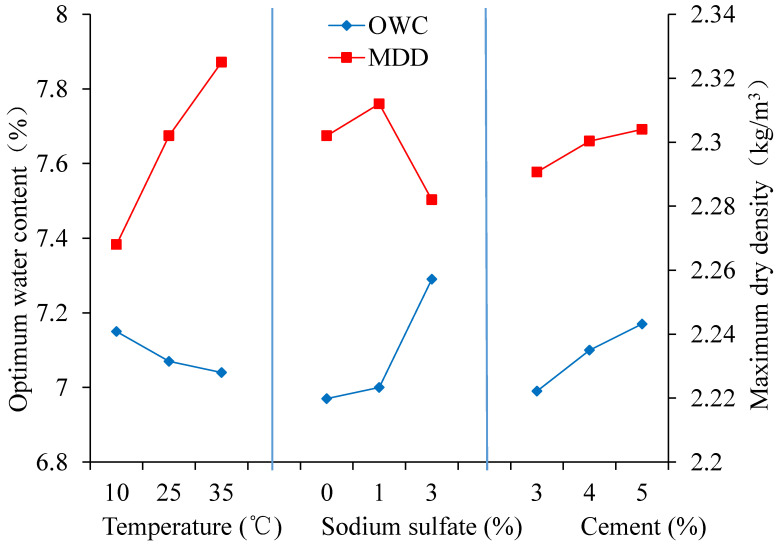
The influence of three factors on the compaction results.

In order to reflect the effect of three factors on the MDD and the OWC of cement-stabilized macadam materials’ compaction test. The level of each factor was regarded as the abscissa, the MDD and the OWC are regarded as the ordinate shown in Figure 17.

Figure 17, shows the relationship between three factors and the compaction results of cement-stabilized macadam materials. The following views can be obtained: (1) the MDD gradually increases with the increase of the temperature, while the OWC gradually decreases. When the temperature is 35 ± 2 °C, the MDD reaches maximum value, and the OWC reaches minimum value. (2) When the content of sodium sulfate changes from 0 to 3%, the MDD first increases and then decreases as the content of sodium sulfate increases, and then reaches the minimum value, but the OWC always increases as the content of sodium sulfate increases, and then reaches the maximum value. (3) Both the MDD and OWC increase as the content of cement increases. When the content of cement is 5%, the MDD and OWC all reach maximum value.

## 5. Conclusions

In this study, the causes of arch expansion section were conducted. On this basis, the compaction characteristics of CSMBM with different cement content, sodium sulfate content and temperature were carried out by the controlling variable method. Then, the effects of the cement content, sodium sulfate content, temperature on the compaction results were analyzed by orthogonal tests. The main conclusions of this study are as follows:

(1) The arch expansion mainly occurs in pavement surface and cement-stabilized macadam base. The temperature, sodium sulfate and cement content may be the main causes of arch expansion. Because there is a lot of soluble salt content in subgrade filler, base materials and water, and the content of SO_4_^2−^ ion is higher than that of Cl^−^, and there is a large difference between maximum temperature and the minimum temperature each month.

(2) The compaction test results are affected by the temperature variation, sodium sulfate content and cement content. With the temperature increasing, the OWC decreases, and the MDD increases due to the proportions of sodium sulfate solution, Na_2_SO_4_·10H_2_O and thenardite affected by the temperature. With the sodium sulfate content increasing, the wetness degree and cohesion of the material decrease, the OWC increases, and the MDD decreases due to the existence of the unreal dry phenomenon. With the cement content increasing, the MDD and the OWC increase due to the water absorbed by cement to form the hydrates to fill the pores.

(3) With the temperature and the cement content increasing, the compactness of the structure composed of aggregates and cementitious material improves gradually due to the columnar Na_2_SO_4_·10H_2_O crystallization and decreasing pores. With the sodium sulfate content increasing, the morphology and structure of cementitious materials are increasingly rough due to the columnar Na_2_SO_4_·10H_2_O crystallization tested by EDS and pores increasing.

(4) The orthogonal test results show that the temperature variation has the greatest influence on the MDD, while the sodium sulfate content has the greatest influence on the OWC. In order to ensure the compaction quality of CSMBM in a sulfate saline soil area, the construction temperature, the sodium sulfate content, and the cement content should be controlled.

## Figures and Tables

**Table 1:** The content of soluble salt, SO_4_^2−^ ion, and Cl^−^ ion in subgrade filler.

Number	Soluble Salt (g/kg)	Cl^−^ (mmol/kg)	SO_4_^2−^ (mmol/kg)	C(Cl^−^)/2C(SO_4_^2−^)
1	3.090	12.901	66.708	0.097
2	7.850	5.775	16.667	0.173
3	11.600	8.225	135.000	0.030
4	10.110	6.620	109.792	0.030
5	8.750	8.507	55.042	0.077

**Table 2:** The content of soluble salt, SO_4_^2−^ ion, and Cl^−^ ion of cement-stabilized macadam base materials in filed.

Number	Soluble Salt (g/kg)	Cl^−^ (mmol/kg)	SO_4_^2−^ (mmol/kg)	C(Cl^−^)/2C(SO_4_^2−^)
1	1.280	2.535	21.667	0.058
2	2.720	11.549	25.833	0.223
3	1.240	1.690	6.250	0.135
4	1.990	2.817	39.167	0.032

**Table 3:** The content of soluble salt, SO_4_^2−^ ion, and Cl^−^ ion in Water.

Number	Soluble Salt(g/L)	Cl^−^ (g/L)	SO_4_^2−^ (g/L)	Type
1	1.765	0.103	0.376	Fresh Water
2	3.787	0.238	0.525	Brackish Water
3	8.767	0.144	0.560	Salt Water

**Table 4:** Percent passing of aggregates.

Screen Size (mm)	Percent Passing (%)
0.075	1.5
0.6	11.5
2.36	22
4.75	27
9.5	48
19	77
31.5	100

**Table 5:** Tap water indexes.

Test Items	PH	Soluble (mg/L)	Cl^–^ (mg/L)	SO_4_^2−^ (mg/L)
Standard	≥4.5	≤10,000	≤3500	≤1500
Results	7.45	213.0	62.63	269.6

**Table 6:** The specific scheme of the tests.

Schemes	Temperature (°C )	Sodium Sulfate Content (%)	Cement Content (%)	Water Content (%)				
1	10	3	3	5	6	7	8	9
	25							
	35							
2	10	0	3	5	6	7	8	9
		1						
		3						
3	10	3	3	5	6	7	8	9
			4					
			5					

**Table 7:** Factor levels in orthogonal tests.

Level	Temperature A (°C)	Sodium Sulfate Content B (%)	Cement Content C (%)
1	10	0	3
2	25	1	4
3	35	3	5

**Table 8:** The mass of each material.

Test Mixture	A (°C)	B (kg)	C (kg)	Aggregates (kg)
1	35	0	0.27	5.4
2	35	0.162	0.216	5.4
3	35	0.054	0.16	5.4
4	25	0	0.216	5.4
5	25	0.054	0.27	5.4
6	25	0.162	0.16	5.4
7	10	0.054	0.216	5.4
8	10	0.162	0.27	5.4
9	10	0	0.16	5.4

**Table 9:** The results for orthogonal test.

Test Mixture	Factors			Maximum Dry Density (kg/m^3^)	Optimum Water Content (%)
	A (°C)	B (%)	C (%)		
1	10 °C	0%	3%	2.276	6.73
2	10 °C	1%	4%	2.271	7.16
3	10 °C	3%	5%	2.265	7.49
4	25 °C	0%	4%	2.301	7.05
5	25 °C	1%	5%	2.318	6.93
6	25 °C	3%	3%	2.280	7.15
7	35 °C	0%	5%	2.322	7.09
8	35 °C	1%	3%	2.335	6.86
9	35 °C	3%	4%	2.329	7.13

**Table 10:** The range analysis results.

Index of Test	Type	A (°C)	B (kg)	C (kg)
MDD (kg/m^3^)	L_i1_	2.271	2.300	2.297
	L_i2_	2.300	2.308	2.300
	L_i3_	2.329	2.291	2.302
	Optimal level	A_3_	B_2_	C_3_
	R_i_	0.058	0.017	0.005
	The order	A > B > C		
OWC (%)	L_i1_	7.13	6.95	6.91
	L_i2_	7.04	6.98	7.11
	L_i3_	7.02	7.25	7.17
	Optimal level	A_1_	B_3_	C_3_
	R_i_	0.10	0.30	0.26
	The order	B > C > A		
